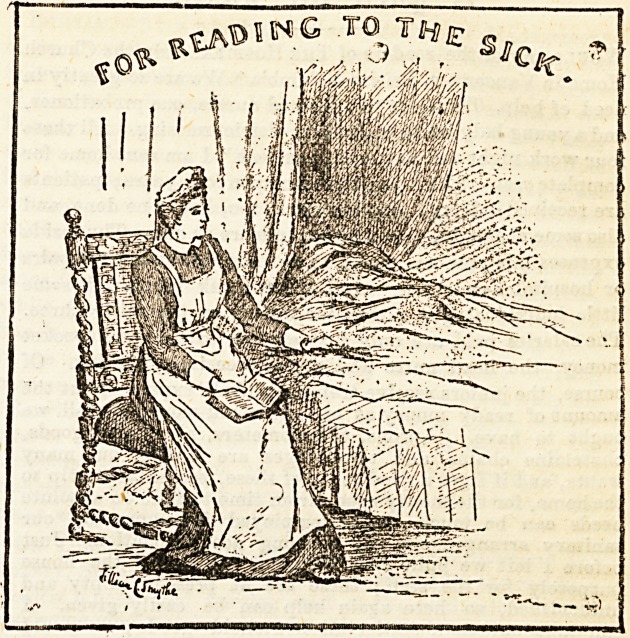# The Hospital Nursing Supplement

**Published:** 1891-07-04

**Authors:** 


### "The Hospital" Nursing Mirror.

Being the Extra Nursing Supplement of "The Hospital" Newspaper.

Contributions for this Supplement should be addressed to the Editor, The Hospital, 140, Strand, London, W.C., and should have the word "Nursing" plainly written in left-hand top corner of the envelope.

### En Passant.

QUEEN'S NURSES IN WALES.—The first annual meeting of the Welsh branch of the Queen Victoria Nurses' Institute was held in the Cardiff Town Hall on June 18th. The Mayor (the Marquess of Bute) presided, and amongst those present were Alderman Lewis, Father Butler, and Dr. Wallace. Dr. Sheen read the first annual report and statement of accounts, which were in every way satisfactory, though more annual subscriptions are needed.

A JUMBLE SALE.—In aid of the Stamford Nursing Society a very successful sale has been held, the Mayor performing the opening ceremony. In the course of his speech the Mayor said the hospitals were limited in number and size, and it would be perfectly impossible for them to receive all the sick people or all the sick poor. There was, therefore, plenty of scope for such institutions as the Stamford Nursing Society, and he was sure all those who required her assistance or advice in this town were well looked after by the lady, whom they termed the Town Nurse. Everybody knew her, and everybody loved her.

NORTH AND SOUTH.—A drawing-room meeting, on behalf of the North London Nursing Association, was held on June 18th, at Avenue House, Finchley, the residence of H. C. Stephens, Esq., M.P. Mr. Stephens introduced Miss Meyer, who would, he said, explain the work of the North London Nursing Association. It was proposed that Finchley should enter into a contract with the Association. The Finchley branch of the Charity Organisation Society had had an offer of £50 towards starting a branch of the Nursing Association in Finchley, and in the first place it was proposed that another £50 should be raised to add to that, and so secure for the parish the right to have one or more nurses from the Association to begin with. A midsummer fête has just be held in aid of the South London Association; there was a large and fashionable attendance, and all went well.

WORCESTER INFIRMARY.—At the late meeting, the Chairman brought forward the subject of the inadequate accommodation for the nursing staff. He said that he was informed that sometimes the dispenser was making up as much medicine for the staff as for the patients. It was not to be particularly wondered at that probationers who spent their life within the walls of that institution should suffer in health. To his mind the remedy was quite plain. They possessed a house called Grove House, close to the infirmary, which might be made into an exceedingly suitable nurses' home. He thought they ought to take that house into their hands as soon as possible and convert it into such a home. Apart from the house, there was a good room outside which could be used for purposes of recreation by nurses who were not on duty, without interfering with others who were taking rest in the house. That would be a better plan than putting up a special building, and he did not think the additional expenditure would be more than £100 a-year, including the rent they would have to sacrifice. The system of nursing at Worcester has been improved and enlarged lately, and we are glad that the Committee recognise their responsibility to the probationers who go to the infirmary to be trained.

SHORT ITEMS.—Ipswich Hospital Committee have decided not to have private nurses to send out.—A bed has been endowed at Caldicote House to the memory of the late Miss Derham.—A patient at Dewsbury Infirmary has been poisoned by the nurse administering a dose of carbolic acid by mistake.—The Queen has presented a portrait of herself to the Training Home of the Edinburgh Jubilee Institute.—In the House of Commons on June 18th, in answer to a question about the R.B.N.A., Sir Michael Hicks Beach said he had been unable to satisfy himself that the means proposed to be adopted by the Association were adequate to carry out its objects. He was not aware of any grounds for reconsidering the matter.—The old quarrel of nuns versus lay nurses is raging in Portugal. The lay nurses have lately been introduced, and of course do not work quite smoothly at first.—Miss Field, who went with Miss Kate Marsden as far as Omsk, has returned to Berlin to plead for funds.

THE SECOND THOUSAND.—Questions are pouring in on us with regard to the reception of the Second Thousand, and as soon as we know definitely the date of the reception we will state it here. In the meanwhile, we should advise members to hold themselves disengaged from July 20th to the end of the month. It is hoped that it will be possible for the Princess of Wales to hold the garden party during those ten days. To save time and trouble will any nurse who knows she will be unable to be present send word to that effect to the Manager of the Pension Fund? "Nurse Ina" writes that she would like to subscribe a small sum to the fund the Princess is raising for Mrs. Grimwood, and says doubtless other nurses would like to do so if they knew where to send. Doubtless any sums sent through Miss Knollys, Marlborough House, S.W., would reach the desired destination. Let every nurse, for instance, who desires to show her sympaty with the Princess of Wales in this movement, send Miss Knollys a postal order for one shilling at once.

A STOCKTON SCANDAL.—A wardman named Dolan has been accused at Stockton of ill-treating a pauper inmate of the infirmary; there are other complaints and rough treatment by Dolan, but he has now left the infirmary. Where the scandal comes in is here; the infirmary holds from 50 to 60 patients; there are two day nurses, no night nurse, and it is during the absence of the nurses that the cases complained of occur. Dr. Horne naturally desires that a night nurse should be appointed, but Mr. G. M. Watson, Chairman, said three nurses for 53 patients was too many. Some day Mr. Watson will be very ill and will doubtless be aware of how long and weary the nights can be; we dare not be cruel enough to desire that he should spend one single night of fever and anguish without skilled attendance, but we do desire that he and the other guardians who voted with him, should seriously consider that they are refusing to the sick and dying the last comforts that they will ask of man. The pauper has in all probability had a hard life of it; it is a terrible responsibility to sentence him also to a hard death. Twelve guardians upheld the Chairman, seven voted against him; we ask the people of Stockton to consider whether those twelve were doing wisely. Every woman in Stockton must know how wearisome it is to nurse one patient, even if that one patient is a dearly loved child; then can it be true that "three nurses to 53 patients is too many"?

### Lectures on Surgical Ward Work and Nursing.

By Alexander Miles, M.B. (Edin.), C.M., F.R.C.S.E.

Lecture XXVIII.—RULES FOR BANDAGING.

There are certain general principles and special rules always to be borne in mind in applying a bandage, and, trifling and unimportant as some of them may appear at first sight, you will do well to pay some attention to them, as much of your success as a bandager, and still more of your patient's comfort, depends on the way in which you appreciate and apply them. 1. If possible, stand in front of your patient in applying a bandage. 2. Never put a bandage next the skin. Always have a layer, however thin, of absorbent wool between the bandage and the skin of the patient. This will prevent the retention of the cutaneous secretions, which, decomposing, cause irritation as well as the chafing and even abrasion of the skin so often induced by hard, non-porous bandages. Sometimes it may be allowable to use domette without wool; for example, when the bandage is only to be left on for a few hours, but when applied for lengthened periods you will be wise to keep by the rule. 3. Never let skin surfaces be opposed. Thus, when the hand or foot is bandaged up, the fingers and toes should be separated by layers of absorbent wool; when the arm is bound to the side, a pad should intervene between it and the chest wall; and in females with pendulous mammæ, the adjacent skin surfaces should be similarly protected. The result of neglecting this precaution is, that the decomposition of the sweat and other skin secretions is the source of irritation which may even set up an inflammation of the skin, going on to superficial ulceration. 4. In bandaging a limb, always place it in the position it is intended to occupy afterwards. By doing so you will avoid the risk of your bandage becoming slack on the one hand or constricting the part on the other. 5. Fix the bandage to begin with. The reason for this is obvious. It is best done by making a figure-of-eight turn round the nearest joint. 6. Apply the bandage from below upwards, and from within outwards, passing over the front of the limb. By proceeding from the distal extremity towards the trunk you avoid engorgement of the limb, which would inevitably happen did you reverse the direction. Passing from within outwards, and over the front of the limb, is rather a matter of convenience than necessity. 7. Use equable pressure throughout. This is most important, as otherwise you will have one part of the limb tightly constricted, leading to congestion and œdema of the part beyond, and all degrees of harm, from slight discomfort up to actual gangrene of a limb, have resulted from want of attention to this rule. Always keep a watch on the tips of the fingers and toes, and, on the appearance of the least œdema or discolouration, remove the bandage at once and reapply it, using more padding or less tension as you find indicated. These precautions are specially necessary in children. 8 Each turn of the bandage should overlap two-thirds of that which preceded it. This helps to ensure equable pressure, gives the bandage a certain amount of rigidity, as well as making it look neat. 9. Keep all the margins parallel, all the crossings and reverses in the same line, and rather towards the outer aspect of the limb. By so doing you will attain to some degree of neatness. 10. Finish the bandage by securely fixing it. This is best done by means of a safety pin, which should always be inserted in the long axis of the bandage, and not across it. Failing a safety pin, the end of the bandage may be slit into two tails, one brought back over the limb and tied in front with the other. This should be tied in a reef bow or knot.

Knots.—In surgery the only knot which is permissible is the square or reef knot, in which both ends of the thread pass in the same direction through each loop, and when tied the loose ends lie parallel with the turns of the bandage. In making it, keep the end which is further from you in making the first turn also the farther away in making the second. The granny-knot is more apt to slip, and the loose ends lie at right angles to the first turn, thus being less neat. The clove hitch is used to fix a patient in the lithotomy position, or to restrain the limbs during an operation. It has the advantage of getting tighter the more it is pulled upon, but of never getting tight enough to injuriously constrict the limb. It is made as follows: "Grasp the bandage with the left hand supine and the right prone, now pronate and supinate the two hands respectively, and slide both loops into the left hand." "Another plan is to make two successive loops in the same direction, and place one behind the other." (Caird and Cathcart.) The surgeon's knot is made by doubling the first turn of a reef knot. It is less likely to slip and become slack while you are making the second turn. It is especially useful in ligaturing blood vessels.

How to Remove a Bandage.—This should be done by simply reversing the manipulations made in applying it. The terminal end should be seized in one hand and then passed behind the limb into the other hand, and so on from one hand to the other as each turn is removed, the loose bandage being gathered evenly into a bundle, and not twisted upon itself. This means of removal facilitates the re-rolling of the bandage, or the washing of it if this be necessary and permissible. Having thus laid down the general principles which are to guide you in applying all forms of bandages, it may be well to describe in some detail a few of the more commonly used bandages, and if the manipulations described be actually gone through by the reader, the descriptions will be very much more easily followed; in fact, without practising the application of the bandages the time spent in reading the directions will be simply wasted.

### Everybody's Opinion.

*[Correspondence on all subjects is invited, but we cannot in any way be responsible for the opinions expressed by our correspondents. No communications can be entertained if the name and address of the correspondent is not given, or unless one side of the paper only be written on.]*

A CORRECTION.

Mr. A. Reade writes from Charing Cross Hospital: On page lxxiii. of the Nursing Supplement to the last number of The Hospital, I read under "Nursing Medals and Certificates," that in 1883, owing to a disagreement at Charing Cross Hospital between the doctors and the St. John's Community, a certain portion of the Sisters and Nurses, headed by Miss Lloyd, seceded, &c. This is a mistake. The Sisters seceded from St. John's House owing to a quarrel with their own Council. There was no disagreement between the doctors and them. At that time St. John's nursed this hospital by contract, and therefore, as soon as these good ladies left St. John's, they naturally had to leave the hospital.

### Appointment.

Dumfries Royal Infirmary.—Miss Isabella Hamilton has been appointed Lady Superintendent of this institution. Miss Hamilton trained at Edinburgh for two years, and then went to Preston as Charge Nurse, ultimately becoming Night Superintendent of Aberdeen Infirmary, and then Sister over Professor Ogston's wards. Miss Hamilton possesses excellent testimonials, and we congratulate her on her steady rise in her profession.

Erratum.—In last week's "Appointment" read "Steyning" for "Stepney."

### Asylum Articles.

### II.—THE ASYLUM DAY.

The nurses rise at six in summer, and at half-past six in winter, and go straight to the dormitories, where they proceed to get the patients up and moved into the wards; the windows are thrown open, and the dormitories and bedrooms left to air. The nurses go to breakfast in two batches, one lot at seven, and the others at half-past seven. The patients breakfast at ten minutes to eight, the nurses waiting on them, and after breakfast those that choose go to the big hall for prayers. The female patients sit on one side of the hall, and the male patients on the other; the chaplain comes in in his surplice, and reads part of the morning service, and a hymn is sung. The congregation usually numbers about 500, and the singing is hearty, and the behaviour of all is quiet and orderly. Directly after prayers regular work commences; some of the patients go to the laundry, those on the male side go to the workshops, and others, under the direction and with the help of the nurses, set to work to clean and dust the whole of the great building. Everything has to be in order by ten o'clock, when the medical officer makes his official round. He has already been round while the patients were at breakfast, and the matron has also made her first visit; but of course absolute order is not demanded before ten a.m. Each nurse gets half an hour to make her bed and put on fresh uniform before the doctor's visit. By eleven o'clock all the nurses' rooms must be fit for inspection. Each nurse has an afternoon a week for thoroughly cleaning her bedroom, on which occasion she is allowed to take a willing patient to help her. The patients have their dinner at 12.30; it has to be nicely served and properly handed, though it is not unfrequently thrown at the nurse's head instead of being gratefully received. The nurses go to dinner at one o'clock and at 1.30. If the weather is fine the patients who are well enough go out in charge of the attendants from two to four o'clock. The Medical Superintendent, accompanied by the Matron, goes round all the wards at 3 p.m. Two afternoons in the week are devoted to bathing the patients in the acute wards, as this is a serious business and needs the unerring supervision of the nurses. The nurses have their tea at 5 and 5.30; the patients have their tea at six—it is a substantial meal and the last one of the day. There is a musical nurse who visits a ward each evening and plays to the patients, and leads a sort of small concert; in the winter there is a weekly dance and a weekly entertainment; and every Sunday evening there is a sacred concert. We ought to have mentioned before that friends are allowed to visit the patients any afternoon except Sundays. At 7.30 the patients go to bed and the day nurses go off duty at eight, and can do what they choose till ten p.m. If they go out they have to leave their keys with the porter; otherwise there are no formalities, no asking of leave. They get their supper when they choose; it is not a set meal.

There are not many night nurses, and they wear no uniform. They are off duty all the morning, and go to bed in the afternoon. The night nurse sits on a raised platform in the middle of the huge dormitory; all around her are beds in which the patients are sleeping. Should any patient "break out" the nurse can call immediate help by ringing an electric bell.

---

A somewhat remarkable case of deafness is reported from Newark, U.S.A., caused by a pea which remained in a young man's ear for nineteen years. After a lapse of this period it was entirely removed by washing out; why this was not done earlier is not stated, for a pea is not altogether a desirable piece of luggage to carry in the ear for even a few minutes.

### "TAKE SHORT VIEWS."

You know how various little maxims are often handed down in families, such as "Don't drink cold water when you're hot," "Never run hard for a train after a hearty meal"; and sometimes, sad to say, such foolish sayings as "Don't swallow a bit of thread; it'll wind round the heart, and stop it." Well, such maxims, if wise, are very helpful. But nearly all of them have to do with our *bodily* health; it is not very often that we are given maxims relating to our *moral* health. Now, the maxim which I want to give you to-day relates to this—to our moral health, and it is this—"Take short views." It has been very helpful to me, and to several people I have known. What can I do better, then, than hand it on to you, in the hope that it will help you also?

I do believe that a very great deal of our unhappiness comes from our taking, not short, but long, views. We can't be satisfied to bear the pain of to-day, but must needs try to bear to-morrow's and the next day's too—yes, and sometimes even that of the years to come—all in a lump. It isn't to-day's pain we mind, but the thought that we may have many days of pain, just as bad, or perhaps worse. It isn't to-day's weakness and weariness that we can't bear, but the knowledge that there will be weakness and weariness in the days to come. Or one of us, perhaps, has seen a loved daughter married, or watched her son's vessel sail away towards the far West, and she is thinking, not of her loneliness *now*, but of the lonelinesss which she will feel day after day, day after day.

Now this seems natural, but yet it is very foolish. We shall certainly have to bear to-morrow's pain when to-morrow comes; so where is the use of trying to bear it to-day too, and making ourselves miserable about what may never come, after all—for who can tell what a day may bring forth? This is common sense, don't you think so? Ah! but we should have something better than common sense to help us—we should have faith. In God's strength we can bear to-day's pain; and ought we not to trust that that strength will be ours in the future too? If we cannot now bear to think of to-morrow's pain, that is because God has not yet given us the strength to bear it. Why should He, when to-morrow has not yet come? "It can bring with it nothing, but He will bear us through." Let us once for all tell ourselves that, and then look no more into the future. He can look ahead for us; we have enly to "take short views," and devote ourselves to the patient bearing of to-day's pain, to-day's trouble for Him. Remember, it was Jesus Christ Himself who said, "Sufficient unto the day is the evil thereof."

### A Plea for Help.

Would any of the readers of The Hospital help the Church Home in Vancouver, British Columbia? We are so greatly in need of help. There are two trained nurses, one probationer, and a young lady being trained for district nursing. All these four work night and day, and "at last" I am sent home for complete rest. The home has been open four years; patients are received indoors; outside work, too, has to be done, and also some charitable work, either indoors or out. The inside expenses this year are met, but nothing left over for repairs or hospital necessaries. And what I ask for now is some little individual gift either for myself or the other three. The salaries paid are only just enough to keep for pocket money, the head nurse and myself receiving nothing. Of course, the juniors receive training and all expenses, but the amount of ready money at our command won't buy all we ought to have. Aprons, thermometers, all rubber goods, chatelaine chains and appendages are among our many wants, and if I get a good stock of these, see what a help to the home, for the money spent from time to time on absolute needs can be much better employed in improving our sanitary arrangements and sleeping accommodation. Just before I left we built four rooms at the top of the house purposely for the staff; these are at present empty and unfurnished, so here again help can be easily given. I can take out as a settler all household goods; so could a nurse, if too poor, beg, and send one present for the "home." Nearly all our inside patients are homeless, and English. We make no difference in rank, all have our care, and we are thankful and very happy in our work. The oft-repeated query, "What should I do without St. Luke's Home?" makes up for the many struggles we have. All nurses know the sad side of life, but do they know what it is to see the patient brought back to life, and when recovering, no money for either nurse or patient? and, as is always the case, the patient's condition and trouble becomes the nurse's chief anxiety. Supposing we know or hear of a patient's relations we write to enclose a charge suitable to their condition in life, but don't forget that till the money reaches us we must provide food and shelter, and we are debarred from leaving our patient to go out and earn money. Our expenses are very heavy, everything costs double if we go outside and earn salaries; we can get along, but if the home is full we must stay indoors, and sometimes I can hardly meet the housekeeping expenses. The doctors help us, also the druggist, or else we should never get on. Can you wonder now, while visiting England, I try to beg and carry back a little of what seems "so cheap"? I am leaving the first week in August, and anything sent to me at the enclosed address, prepaid, will be very thankfully accepted. One particular donation I shall be glad of: Two nurses' black cloaks—one for winter and one for warm weather. The Bishop of New Westminster and our own Rector (the Rev. H. G. Fiennes-Clinton) endorse my appeal. Any money sent can be placed to the St. Luke's Home account, Bank of Montreal, London. How very much I hope someone among you will help us; and please remember we thankfully receive anything for household or hospital.

Frances, Sister-in-Charge.

(Now staying at Corgrig, High Park, Ryde, Isle of Wight.)

### Wants and Workers.

[Under this heading, we propose to try whether we can be useful to our readers in making the wants of some known to others who are willing to do what work they can to aid the great cause of curing and cheering the sick. Wants can only be inserted from those who are connected with some institution or association, or who are willing to have their full name and address printed.]

*Nurse Winter* wishes to thank Miss Summers, Mrs. Price, "A Reader of The Hospital," and "As Requested" for responding to her want printed on June 13th.

*Miss Wilson*, Hon. Secretary of the Workhouse Infirmary Nursing Association, 6, Adam Street, Strand, will be very glad of *R.P.*'s offer of help with writing. Will *R.P.* kindly send her address to Miss Wilson early in August?

*Clothes Wanted.*—Would any one be so kind as to send a few old children's clothes to a small home for children, or to do a little needlework for them? The material supplied if necessary. Anything gladly received by the Matron, Melicent House, Sandown, I. of Wight.

### The Nurses' Wedding Gift.

All our readers know how Miss Durham proposed long since in our pages that the nurses should give a wedding present to Princess Louise of Schleswig-Holstein, as a mark of their appreciation of the interest her Royal mother, Princess Christian, has ever taken in nursing. Princess Christian had herself a silver wedding coming off, but it was felt that the indirect compliment through the daughter would be more modest and pleasing; the young enjoy the receiving of gifts much more than those who, like Princess Christian, must possess more trinkets than they know what to do with. So Miss Durham undertook to receive subscriptions, which were limited to a very low sum, but the nurses of the United Kingdom responded in such numbers that over £20 was collected in the end. Then a committee was formed consisting of Miss Durham, Miss Pigott, Matron of Preston Infirmary, Miss Raynor, and Dr. George W. Potter, and after approaching Princess Christian to find out what her daughter would like, a diamond crescent was bought. But Miss Durham has the advantage of being constantly in attendance on the Poet Laureate, and with his usual gracious courtesy, Lord Tennyson undertook to write a short verse for the occasion. Messrs. Macmillan and Co., who are Tennyson's publishers, gave copies of all Tennyson's poems on hand-made paper, and the committee had these volumes handsomely bound in vellum.

Such was the history of the movement, the fulfilment of its aim was seen on Friday, June 26th, when a deputation waited on Princess Louise of Schleswig-Holstein at Buckingham Palace and made the presentation. The deputation consisted of Miss Emma Durham, Miss Pigott (of Preston), Miss Sprigg (of the New Bond Street Institute), Sister Marie Rees (of the Devonshire Square Institute), Miss Raynor, and Miss Smythe. The presentation took place in an antechamber of the central hall, and the deputation was introduced by Colonel Elliot. Her Royal Highness Princess Christian, followed by Her Highness Princess Louise Augusta, entered the room punctually at four o'clock and shook hands with the assembled nurses. Miss Durham then stepped forward, and handing the volume in which was Lord Tennyson's inscription, desired Princess Louise to accept the gift and the good wishes of all the nurses of the kingdom. Miss Pigott then presented the diamond crescent. Princess Louise thanked the deputation, and expressed herself greatly pleased with the gift. It was pretty to see how girlish and nervous the daughter was, leaving Princess Christian to take the lead, which she promptly did in the most kindly manner. Everything was done very simply, which was just what the nurses wanted. After Princess Christian and Princess Louise had retired, Colonel Elliot showed the deputation through the State rooms of the Palace. Miss Durham and Sister Marie Rees were the only two nurses in uniform, but Miss Durham was wearing the Royal Red Cross, the Soudan medal, and the badge of the Stafford House Committee. The verse written by Lord Tennyson was as follows:—

"Take, lady, what your loyal nurses give,
Their full God-bless-you with this book of song;
And may the life which heart in heart you live
With him you love be cloudless and be long."

Princess Louise has graciously given permission to have the page on which the inscription is written photographed, for it is believed that many nurses who subscribed to the gift would like to have the facsimile of the Laureate's writing. Miss Durham wishes us to express her thanks to all her fellow workers for their help in getting the present. Our readers will be interested to learn that Miss Durham goes shortly to Shanklin to take charge of a Surgical Home for Children.

### The Lords' Committee.

ONE sentence of Miss Twining's evidence, omitted last week, must be recorded as of special interest to nurses. "In my opinion the matron should have more undivided authority over the nurses (in union infirmaries), as I see great evils arising from the medical officers being in supreme command."

Mr. T. D. Mann, clerk to the Metropolitan Asylums Board, said the Board was created in 1867, and consisted of seventy-two members. They had 2,429 beds for small-pox in the fever hospitals, and 1,150 in the ships. They had no power to detain any person whose friends desired to remove them before they were fit to leave the hospital. The patients were received on the medical officers' certificates, and the Board looked to the local authorities for payment, they being able, in their turn, to recover the money from those able to pay. During the ten years ending 1889, they treated 34,433 fever cases, and 26,357 small-pox cases. The average cost varied from 2s. 6d. per patient at the Northern Convalescent Hospital, to 4s. 4d. at the South Western. Each hospital was governed by a separate committee, which appointed the nurses, who were under the control of the matron. They did not train nurses nor give certificates.

Mr. W. T. Howard, clerk to the Bethnal Green Board of Guardians, admitted that the infirmary had been overcrowded, and stated that the guardians had been unable to find a suitable site for a new building.

Mr. W. Vallance, clerk to the Whitechapel Board of Guardians, considered that the present system of poor-law relief in London adequately met the case of the destitute sick. He favoured admitting students to the infirmaries, but he understood the poor preferred the infirmary to the London Hospital, because there were no students at the infirmary.

Mr. T. B. Campbell, secretary to the Royal Westminster Ophthalmic Hospital, said the hospital was entirely free, and no letters of recommendation were required. The income last year was £2,272, and the expenditure £2,169.

Mr. F. Andrew, Secretary of the Royal Hospital for Incurables, Putney, stated that there were 218 inmates at the institution, of whom 38 were men. There was no resident medical officer, but they had a medical man whose duty it was to visit the hospital every week-day, and at any time when specially called. The expenditure on the hospital was £12,999 annually, of the seaside home £1,097, and in pensions £11,129, that on management bringing the total up to £28,000. The receipts last year were £44,109. The Matron was responsible to the Committee, but had full control of the house. There had been complaints from time to time about the Matron and the diet, but no specific cases were ever mentioned, and no names were given, so that it was very difficult to deal with them. It was true that some one wrote to the Prince of Wales to prevent his laying the foundation stone of a new building, but the complaint was investigated by the Archbishop of Canterbury, with the result that the Prince kep his appointment. There had also been some correspondence with the Duke of Portland, to whom the Committee sent a complete, and, as it believed. a satisfactory answer, but no further reply was received, In answer to Lord Cathcart, witness said there would not be any advantage in having a medical officer to check the Matron, nor would there be enough work for him to do. Lord Cathcart: Not for 218 patients? Certainly not. The present arrangement works in every way satisfactory.—Lord Cathcart: For the Committee and Matron, perhaps? I object to the Matron being spoken of in that invidious way. The members of the Committee have eyes.—Lord Kimberley: The question was perfectly admissible, and if you want to know, the impression your evidence has given me it is that everything connected with the Matron is exceedingly unsatisfactory.—Mr. G. Browne, Chairman of the General Practitioners' Union, complained of the harm hospitals did the general practitioner, and gave some cases in point.

### Presentations.

BUNGAY.—At a meeting of the Committee of Management of the above Institute, held this 19th day of June, 1891, the following resolution was unanimously passed, and a copy thereof directed to be sent to Nurse Emily: "The Trustees of the Bungay Nursing Institute, while congratulating Nurse Emily on her appointment as Matron of the hospital to Llandudno, feel they cannot allow her to leave without expressing great satisfaction at the admirable manner in which she has carried out her duties as nurse during the four years the Institute has been established. Her great kindness and unremitting attention to the poor has won for her their love and gratitude, while the tact and energy displayed in carrying on her work has done much to make the Institute a success." The poor of the town have given Nurse Emily a handsome dressing case and hand-bag. The former bore a silver plate with the following inscription: "Presented to Nurse Emily by the poor of Bungay, June 19th, 1891, as a mark of esteem for her kindness towards them as the nurse of the Institute at Bungay." Nurse Emily expresses her warmest thanks to the many who so kindly contributed thereto.

ON Thursday, the 4th inst., at the Chester General Infirmary, Sister Lucy S. Gane was presented by the resident medical officers and nursing staff of the institution with a handsome gold watch and chain, the occasion being her departure from the institution, after a connection of over ten years, to take up district nursing at Paddiham, Lancs.

ON June 13th a pleasing presentation took place in the Board-room of the Wigan Infirmary, when the nurses of the institution asked Miss Macintyre, the Matron, to accept a small rosewood table as a token of their esteem. Nurse Valentine made the presentation, and said that the nurses could not but remember the kindnesses they had received at the hands of Miss Macintyre during the twelve months that she had now been at the Wigan Infirmary. It was to signify their respect for her that the nurses had decided to make a gift, and they hoped that the same happy relations between themselves and the Matron would continue in the future as in the past. Miss Macintyre said she could not thank the nurses enough for their gift, and she trusted that she would long continue to have their confidence. It was her desire to bring up the nursing staff at the Infirmary to such a pitch of excellence that they would be second to none in the kingdom.

### Notes and Queries.

TO CORRESPONDENTS.—1. Questions or answers may be written on post-cards. 2. Advertisements in disguise are inadmissible. 3. In answering a query please quote the number. 4. A private answer can only be sent in urgent cases, and then a stamped addressed envelope must be enclosed. 5. Every communication must be accompanied by the writer's full name and address, not necessarily for publication. 6. Correspondents are requested to help their fellow nurses by answering such queries as they can.

### Answers.

*Anxious.*—The dolls are on view at this office (140, Strand) between the hours of 10 a.m. and 4 p.m.

*Burman.*—You had better get "The Englishwoman's Year-book," price 1s., published by Hatchard, for full particulars. The four years' course at the London School of Medicine for Women is £80 for lectures and £45 for hospital practice. The Edinburgh School of Medicine for Women, also a four years' course, £80. Also women are admitted to the ordinary classes of the College of Surgeons, Dublin. The matriculation of the London University answers as a preliminary examination. In all, you can reckon that £400 is the very least on which a woman can become a doctor.

*C.W.S.*—We do not know Miss Meyrick's address. Perhaps, if she sees this she will send it us and then we will forward it.

*M.M.*—You need not hold a certificate to be a Queen's nurse, but you must have trained for one year in a good hospital. Age has nothing to do with it.

*H.T.C.*—We never prescribe.

*W.H.P.*—We never prescribe; nor is it likely any of our readers could help you. You seem to have had the best advice possible; we can only sympathise with you.

*Private Monthly Nurse.*—Some of the Queen Charlotte's nurses attended the garden party last June in in-door uniform; but there is no reason why you shouldn't wear the bonnet and cloak you propose over your white dress. The date will probably be late in July, but will be stated definitely here as soon as possible.

*A.E.S.*—We absolutely refuse to print wholesale attacks on officers of institutions; supposing one house surgeon is careless, doubtless there is one matron who is cross. Do try to bear and forbear.

### Away from the Wards.

"You are sure to lose your way."

"Never mind; send the crier after us."

"He will lose *his* way."

Our fellow-nurses at St. Agatha's crowd round us with warnings wise and foolish, but if the spirit of adventure was a grand and beautiful thing in Drake and Columbus, why should it be otherwise than praiseworthy in two independent pros? We are tired of the beaten tracks, and longing for paths unknown, and so, in spite of ominous looks, we grasp our umbrellas and depart to Liverpool Street, en route for Chingford. We have arranged to walk through the intricate forest paths to Epping, a distance of seven miles, and carry with us the written directions of a friend acquainted with the neighbourhood. The train puts us down at Chingford, and turning our backs on the village, we walk to an old barn which is the starting point of a broad track called the Green Ride. As we pass into the forest, the sky clouds over, and rain-drops patter fast, but we decide that it is but a shower, and keep bravely on down the sloping road. Presently we pass a small lake and come to a solitary oak, around which a seat has been placed. By this time the rain is over and the sun is shining. We stay to look at a briar-rose that spreads its hundreds of pink petals to the sun; the rain-drops glitter in its amber hearts and its delicate perfume is all the sweeter for the wet. The cuckoo, his notes a little rusty, is calling in the distance, and up in High Beech Wood, rising at the end of our path, the blackbirds and thrushes are apparently holding parliament, for they are rather chattering than singing. After a little while the track takes a dip into a deep hollow filled with pollards and a luxuriant growth of giant bracken fern, and further on we find another lonely oak, with its convenient bench. Now the paths are getting mixed and we are forced into a consultation with our paper guide, which ends by a plunge into a gravel-pit, and a scramble along a road that would have rejoiced the heart of a pilgrim of old, though it causes us to make undignified remarks when we find the hard gravelly mud is scratching our nice new walking boots in the most inhospitable way. After making excursions into wrong paths once or twice we come again to a green ride, which descends a little, bringing us to a lovely forest view. Beneath is a thickly-wooded valley, from which rises a steep, pointed hill, covered densely with foliage. Around are other hillsides closing in the hollow.

In the middle of our track we discover an artist engaged in depicting this charming but difficult scene. He is young and mild and seems quite pleased to see us; man is a gregarious animal even should he be a genius. Observing his affable expression we stay to admire the sketch, which is not all it might be, and say a few encouraging things about shadows and schemes of colour which we remember mostly from an article we have read. He is grieved to part with us, and gazes after us regretfully as we go down hill. Taking a narrow path to the right we cross the valley and ascend the slope on the opposite side. Here we inadvertently sit down to rest on an ant hill but do not stay long.

There are banks and mounds just here over-grown with polled oaks and commanding a beautiful view; they look like the old British earthworks that we have seen at Whittingham and other places. We find a convenient stump and sit back to back on it, while we eat a sandwich. We saw a brook in the hollow, but the water was not delectable, so we put off being thirsty till we reach civilization. Then we go back to our little path which becomes most difficult to trace and cross some more rough ground and an open rushy space. The path broadens, but narrows again, and as we descend towards Little Monks Wood, a low hill covered with beeches, the ground is carpeted with a kind of heath and our serge skirts are fluffy with the soft down of the cotton-plant. There is a smell of honeysuckle in the air, and we see some of the fragrant clusters on the top of a neighbouring bush. The great humble bee is darting frantically about, and his sleepy buzzing makes us feel lazy, but we shake ourselves and plod through the beeches until we cross a little stream and find ourselves in Great Monks Wood. No path is to be seen, and we wander about a long time before we catch sight of a keeper's lodge, which we know is one of the landmarks. Again we get into a broad green ride, which is a name given to any of the larger forest paths, and descending into a glade, find the opposite bank golden with the late flowers of the broom. Up we climb again through bushes of glossy holly and graceful silver birches, and away to the right of Theydon Wood, into Epping Thicks, where a lovely avenue of beech and oak casts a grateful shade. Some of these trees are very old. The feathery bracken and yellow gorse grow thickly on either side. Presently we come to a road from which charming views can be seen, and now our adventures are over, for we have only to walk on to the straggling little village of Epping, where we find a clean inn called the Thatched House, and enjoy a high tea in the small best parlour. Then we go to the station and take tickets for Liverpool Street, well satisfied with ourselves, and with all men—I mean women. And at night, in the long domitory, the nurses gather round us, to hear of our adventures in the trackless forest, and to share our treasure of moss and fern, which will brighten the wards for a week to come.

### Amusements and Relaxation.

### SPECIAL NOTICE TO CORRESPONDENTS.

**Third Quarterly Word Competition commences July 4th, 1891, ends September 26th, 1891.**

Competitors can enter for *all* quarterly competitions, but no competitor can take more than *one first prize* or *two* prizes of any kind during the year.

Proper names, abbreviations, foreign words, words of less than four letters, and repetitions are barred; plurals, and past and present participles of verbs, are allowed. Nuttall's *Standard* dictionary only to be used.

N.B.—Word dissections must be sent in WEEKLY not later than the first post on Thursday to the *Prize Editor*, 140, Strand, W.C., arranged alphabetically, with correct total affixed.

The word for dissection for this, the FIRST week of the quarter, being

"HAYFIELD."

| Names. | June 25th. | Totals. | Names. | June 25th. | Totals. |
|---|---|---|---|---|---|
| Christie | 27 | 407 | Ivanhoe | 23 | 349 |
| Patience | — | 214 | Weta | — | 147 |
| Agamemnon | — | 367 | Lady Betty | — | — |
| Hope | 29 | 414 | Mortal | — | 76 |
| Reldas | 25 | 410 | Little Eliza | — | 147 |
| Lightowlers | — | — | Dove | — | 95 |
| Nurse J. S. | 27 | 356 | Ladybird | — | 141 |
| Qu'appelle | — | 170 | Psyche | 24 | 358 |
| Jenny Wren | 22 | 347 | Ugug | — | 229 |
| Wyameris | 25 | 409 | Harrie | — | 69 |
| Paignton | 24 | 346 | Grannie | 19 | 336 |
| Theta | — | — | Eale | — | 169 |
| Success | — | 17 | Grimalkin | — | 53 |
| Tired | — | 136 | Nurse G. P. | 16 | 125 |
| M. G. | — | 188 | | | |

### Notices to Correspondents.

Results of Second Quarterly Word Competition will be published next week.

N.B.—Each paper must be signed by the author with his or her real name and address. A *nom de plume* may be added if the writer does not desire to be referred to by us by his real name. In the case of all prize-winners, however, the real name and address will be published.

All letters referring to this page which do not arrive at 140, Strand, London, W.C., by the *first post on Thursdays*, and are not addressed PRIZE EDITOR, will in future be disqualified and disregarded.

## Figures and Tables

**Figure f1:**